# DETECTING CRYPTIC INDIRECT GENETIC EFFECTS

**DOI:** 10.1111/evo.12401

**Published:** 2014-05-05

**Authors:** Nathan W Bailey, Jessica L Hoskins

**Affiliations:** 1 Centre for Biological Diversity, University of St AndrewsSt Andrews, Fife KY16 9TH, United Kingdom; 3 School of Biological Sciences, Monash UniversityMelbourne, Victoria 3800, Australia

**Keywords:** *Drosophila melanogaster*, interacting phenotype, interaction coefficient, phenotypic plasticity, social evolution, social flexibility

## Abstract

Indirect genetic effects (IGEs) occur when genes expressed in one individual alter the phenotype of an interacting partner. IGEs can dramatically affect the expression and evolution of social traits. However, the interacting phenotype(s) through which they are transmitted are often unknown, or cryptic, and their detection would enhance our ability to accurately predict evolutionary change. To illustrate this challenge and possible solutions to it, we assayed male leg-tapping behavior using inbred lines of *Drosophila melanogaster* paired with a common focal male strain. The expression of tapping in focal males was dependent on the genotype of their interacting partner, but this strong IGE was cryptic. Using a multiple-regression approach, we identified male startle response as a candidate interacting phenotype: the longer it took interacting males to settle after being startled, the less focal males tapped them. A genome-wide association analysis identified approximately a dozen candidate protein-coding genes potentially underlying the IGE, of which the most significant was *slowpoke*. Our methodological framework provides information about candidate phenotypes and candidate single-nucleotide polymorphisms that underpin a strong yet cryptic IGE. We discuss how this approach can facilitate the detection of cryptic IGEs contributing to unusual evolutionary dynamics in other study systems.

Our understanding of how social traits evolve has historically been afflicted by unique challenges (West-Eberhard 1989; Baldwin 1896). Chief among these is the fact that when two individual animals interact, the expression of a trait involved in the social interaction such as a behavior, a physiological response, or a morphological change, may depend on the phenotypic value of a trait expressed by the interacting partner. Defining an individual's phenotype then becomes problematic because it is not a property of just a single individual. This complicates our understanding of how interacting phenotypes evolve: if the environment consists of other individuals, then environmental effects on trait expression are likely to be underpinned by heritable genetic variation (Wcislo 1989). The environment can therefore evolve, causing feedback that impacts the genetic architecture of social traits, their responses to selection, and selection itself (Moore et al., 1997; Wolf et al., 1999; McGlothlin et al., 2010).

Theoreticians have employed a variety of approaches to model how the evolutionary dynamics of interacting traits differ from those of other traits, and what the likely consequences are for social evolution (Bailey 2012). One class of models capitalizes on a quantitative genetic framework to describe how genes expressed in one individual contribute to the trait expression of another individual (Moore et al., 1997). Such indirect genetic effects (IGEs) turn out to have potentially major impacts on the evolution of traits implicated in a broad array of evolutionary processes, including sexual conflict (Moore and Pizzari 2005), sexual selection (Bailey and Moore 2012), social dominance (Wilson et al., 2011), and aggression (Rodenburg et al., 2008). However, to clearly predict and infer how IGEs affect the evolution of interacting phenotypes, it is necessary to accurately quantify IGEs, their likelihood, the phenotypes involved, and the relative importance of social flexibility compared to other sources of phenotypic plasticity (McGlothlin and Brodie 2009; Bijma 2010).

Two approaches have been used to estimate IGEs. The first, a variance-partitioning approach, divides phenotypic variation into direct and indirect genetic components. This approach originated with early models by Griffing (1967) has been profitably used to estimate the relative magnitude of direct versus indirect genetic variance for male display traits in *Drosophila serrata* (Petfield et al., 2005), and has been adopted by animal breeders (Bijma et al., 2007).

The second approach is trait based and was developed by Moore et al. (1997). Trait-based estimates of IGEs seek to understand how specific trait values in an interacting partner alter the phenotype of a focal individual (Fig.1A). If the genotype of focal individuals is held constant whereas the genotypes of interacting partners are allowed to vary, focal phenotypes can be regressed on interacting partner phenotypes. The resulting partial regression coefficient associated with the phenotypes, ψ, provides an estimate of the magnitude and direction of any IGEs (Moore et al., 1997; Bleakley et al., 2010).

**Figure 1:**
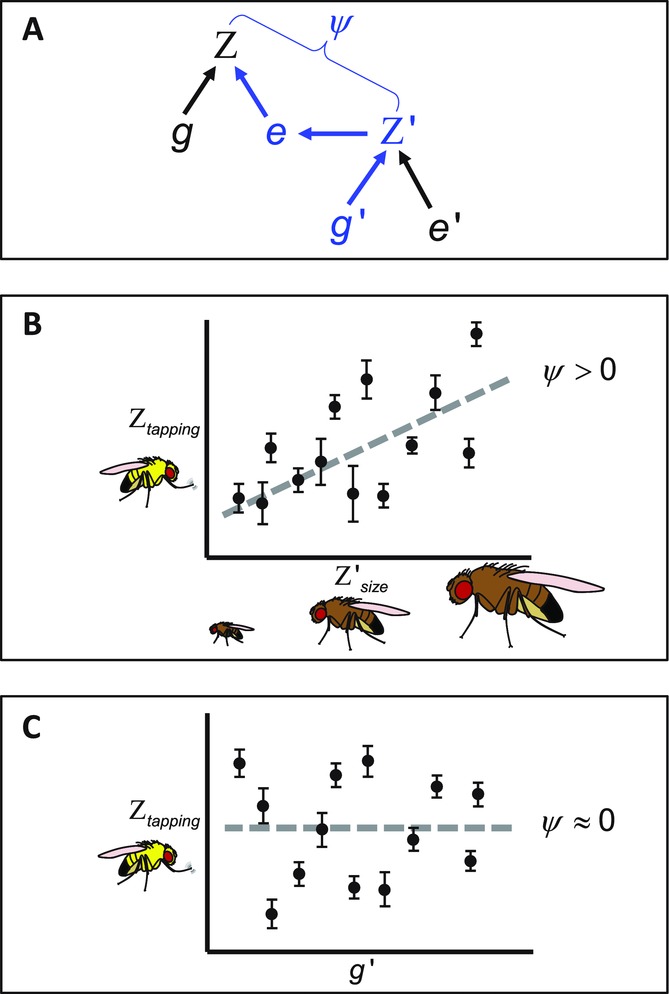
Cryptic indirect genetic effects (IGEs). (A) The traditional path diagram illustrating IGEs that occur when the phenotype of one individual (Z) is affected by the phenotype of an interacting conspecific, denoted by the prime (Z'). The strength of the indirect effect is scaled by the interaction coefficient, ψ, which can be estimated by regressing the standardized focal phenotype on the standardized interacting partner phenotype. The elements in blue trace the IGE: an IGE will only occur if variation in genes expressed in the interacting individual (g') has a causal influence on variation in expression of the trait in the focal individual, via its social environment e. (B) A hypothetical example of an IGE affecting tapping behavior (Z_tapping_) in a focal male *Drosophila melanogaster* strain. In this fictional scenario, as body size of the interacting partner (Z'_size_) increases, focal males exhibit more tapping behavior, and therefore 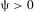. Assuming additive genetic variation exists for body size, this IGE might cause unusual dynamics in the evolution of either or both of the two traits. (C) Another hypothetical example illustrating a strong but “cryptic” IGE. In this scenario, there is clear variation in tapping behavior (Z_tapping_) of the focal male strain, depending on the genotype (g') of the interacting partner. Because the focal strain remains constant, it is straightforward to use a variance-partitioning approach to test whether the interacting partner genotype significantly influences Z_tapping_ (Griffing 1981; Bijma et al., 2007; McGlothlin et al., 2010). However, the interacting *phenotype* is unknown, or cryptic, and it is therefore challenging to determine which phenotypic trait(s) are subject to evolutionary feedback caused by the IGE. Regressing the focal male phenotype (Z_tapping_) on an interacting male phenotype selected by the experimenter could erroneously lead to the conclusion that there are no IGEs for the trait, when in fact 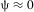 only for the given interacting phenotype under consideration. Thus, trait-based approaches can indicate whether previously specified traits have potential to experience unusual evolutionary dynamics as a result of IGEs, variance-partitioning approaches can indicate whether IGEs are likely to be important, but when there is a strong signature of trait expression dependent on the genotype of interacting partners, it can be a considerable challenge to detect the phenotypic trait(s) that are causally implicated.

The interaction coefficient ψ plays a large role in determining the evolutionary consequences of IGEs: when ψ is large in absolute terms, the rate of evolution of interacting phenotypes can be significantly increased or decreased, depending on its sign (Moore et al., 1997). For example, when IGEs are strong and positive, our expectations for the genetic architecture of traits such as sexual ornaments and associated preferences may change (Bailey and Moore 2012). It is also possible for ψ to evolve as a trait itself, causing further unexpected feedback (Kazancioğlu et al. 2012). The trait-based approach is particularly useful if an experimenter is interested in the potential for previously identified phenotypes to influence each other's evolution through IGEs.

Despite theoretical and empirical developments, a challenge remains for researchers who study interacting phenotypes, and that is the fact that IGEs can elude detection despite being potentially very strong and very important for the evolution of social traits. Figure1 illustrates this challenge using hypothetical examples in *Drosophila melanogaster*. The problem is that empirical approaches for quantifying IGEs might not identify the causative interacting *phenotype* that contributes IGEs to the expression of a focal trait. For example, one might suspect that an individual's dominance status is related to the aggressiveness of its interacting partner—it is reasonable to suspect that fighting with a more aggressive partner would decrease the probability of a focal male emerging as socially dominant (Logue et al., 2010; Wilson et al., 2011). However, this is not a foregone conclusion. Dominance status could depend on any number of hypothetical attributes of the interacting partner, including size, color, pheromones, or physical ornamentation (e.g., Schuett 1997; Kortet and Hedrick 2005; Rhodes and Schlupp 2012). Thus, IGEs affecting dominance status might, in this manner, be cryptic.

If the objective of a study is to use an IGE framework to clarify the evolutionary dynamics of a focal trait that is socially flexible, then it becomes important to identify the main interacting phenotypes that contribute IGEs to its expression. The importance of identifying cryptic IGEs lies in the potentially different evolutionary predictions that arise depending on the traits involved. There is a conceptual parallel with cryptic female choice, which is notoriously difficult to distinguish from sperm competition: a pattern of biased paternity may be observed after a female mates multiply, but detecting whether the biased paternity arises as a result of differences in the ability of males’ ejaculates to compete against one another, or as a result of sperm selection by the female, can be very difficult to disentangle. Nevertheless, the two processes have potentially distinct evolutionary consequences (Eberhard 1996). Cryptic IGEs may be particularly problematic in animal breeding programs, for example, when phenotypes such as crop yield or cannibalism are targeted for improvement (Denison et al., 2003; Rodenburg et al., 2008), or in artificial selection and experimental evolution studies where interacting phenotypes might influence long-term evolutionary trajectories nonintuitively (Poltak and Cooper 2011).

The present study explores the problem of cryptic IGEs and how to detect them. We had two objectives. The first was to illustrate a cryptic IGE using a behavior in *D. melanogaster* that is readily observed and occurs exclusively in the context of an interaction—male tapping. Tapping occurs when a male brings the tarsus of a foreleg into contact with another individual (Spieth 1952; Cobb et al., 1985). It appears to be mostly performed by males, is considered to be a component of the typical male courtship repertoire, and might have chemosensory functions (Rendel 1945; Spieth 1949; Nayak and Singh 1983; Yamamoto and Nakano 1999). Tapping also occurs between males, in which case it may represent misdirected courtship, sampling behavior, same-sex sexual behavior, or aggression (Bailey et al., 2013). We specifically focused on tapping that occurred between males in this study because it is obvious and easy to score.

Having established and validated a strong IGE affecting tapping behavior in the first part of the study, our task was complicated by the fact that it was cryptic as described above. The second objective was therefore to characterize the phenotype(s) underlying the IGE, and we applied two approaches. We identified potential interacting phenotypes using a regression-based analysis that capitalized on publically available phenotype information. We followed up with a genomic association analysis that circumvented phenotypes to directly identify genes that might influence IGEs for tapping behavior. Our results provide a first step toward characterizing a cryptic IGE for male tapping behavior, on both the level of the phenotype and on the level of the genotype. The approach we took appears to be feasible in a number of model and nonmodel systems for which genomic or pedigree information, plus rich phenotypic datasets, is readily available, and its application to other systems has the potential to enhance our ability to predict and characterize the contribution of IGEs to social evolution.

## Methods

### FLY STOCKS AND MAINTENANCE

Behavioral trials were performed using inbred *D. melanogaster* lines and one *D. melanogaster* laboratory strain with a yellow-body mutation. Fifty inbred lines were selected at random from the Drosophila Genetic Reference Panel (DGRP; Mackay et al., 2012); their identities are given in Table S1. The DGRP is a publicly available resource consisting of nearly 200 fully genome-sequenced lines (Mackay et al., 2012). The lines were originally generated by Mackay et al. (2012), by performing 20 generations of full-sib matings using mated females collected from an outbred population in Raleigh, North Carolina. The sib-mating regime yielded an estimated inbreeding coefficient of *F* = 0.986, and the lines can be considered to represent a sample of genotypes present in the wild population (Mackay et al., 2012). In our experiment, males from 50 of these inbred lines (hereafter referred to as “DGRP lines”) were tested in behavioral trials with a strain carrying the yellow-body color mutation, allowing easy identification of each interacting partner. The yellow-body strain was on a wild-type background, Hmr^2^, obtained from the Bloomington Stock Center; FlyBase ID: FBal0144828 (Hutter and Ashburner 1987).

Stock flies were kept at a density of roughly 50 adults in 25 × 95 mm vials, on yeast-seeded cornmeal/agar medium at 18°C. Flies used in experiments were maintained in larger vials (29 × 95 mm) at 23°C on a 12:12 light:dark cycle. We ensured that all males were virgin by collecting them a maximum of 12 h posteclosion under light CO_2_ anesthesia. They were then kept individually in small (16 × 95 mm) vials until use in behavioral trials.

### BEHAVIORAL TRIALS

Our decision to focus on tapping in this study was driven by the fact that it represents a discrete behavior that can only occur in the context of an interaction, it has putative courtship and aggression functions, and it was straightforward to observe and record (Spieth 1974; Cobb et al., 1985). For each interacting individual, we also recorded orienting, following, licking, courtship singing, and abdomen curling in male–male encounters, plus an overall measure of activity level obtained by summing the total number of behaviors of any type over the entire trial. Terminology follows Bailey et al. (2013), and supplemental videos in the same publication show annotated examples of each behavior.

We performed 2000 behavioral trials using socially naïve flies. Five trials were discarded from further consideration after it was discovered they were recorded at too low a temperature (about 17°C). The remaining 1995 trials were performed between 19.4°C and 24.9°C, and between roughly 08:00 and 13:00, to minimize variation in behavior arising from the time of day of observations. Observations were performed in small (16 × 95 mm) vials oriented horizontally using an interval sampling procedure (Bailey et al., 2013). Three evenly spaced, 1-min observations were performed on five pairs of interacting flies at a time, yielding a total of 3 min of observation for each pair. The same observer performed all observations. The occurrence of all the above behaviors was recorded for each male over the 3-min trial period, resulting in a binary measurement of whether each male in a pair performed a given behavior. We focused on the incidence of behavior, rather than intensity, owing to the difficulty of quantifying intensity in behaviors that occur infrequently such as tapping (Bailey et al., 2013). Forty trials were performed for each inbred line.

During each trial, a virgin, 3- to 5-day-old, yellow-body male was paired with a virgin, 6- to 8-day-old male from one of the 50 DGRP lines. Mature males were used to avoid adverse behavioral interactions arising due to the lack of sex-specific cuticular hydrocarbon deposition that has been observed shortly after emergence (Curcillo and Tompkins 1987). The yellow-body males are hereafter referred to as “focal males,” and the inbred DGRP line males as “interacting males.” Although the yellow-body mutation might be expected to cause pleiotropic effects on courtship and other behaviors (Bastock 1956), this would not have confounded the experiment because all focal males were the same yellow-body strain. In addition, although it is not possible to rule out completely, we previously found no evidence to suggest that the yellow mutation dramatically changes the behavior of interacting partners above and beyond what they would exhibit paired with a wild-type fly (Bailey et al., 2013).

### BLIND VALIDATIONS

Due to logistical constraints, we were unable to test all 50 DGRP lines at the same time. We therefore performed a blind validation by re-testing eight of the lines (see Table S1 for details of lines used in the validation). Experimenters were naïve to the incidence of tapping behavior expressed by each. The blind validation block was performed as before. It thus tested whether the tendency of focal males to modify their tapping behavior depending on the interacting male line was repeatable.

The analysis described below recovered an IGE on male tapping behavior related to the startle response of interacting partners. We found this result using publicly available phenotype information for the DGRP (Mackay et al., 2012). Phenotypes quantified in different laboratories can be susceptible to interlaboratory variation caused by unaccounted methodological differences or other environmental effects (e.g., Crabbe et al., 1999), so we performed a small validation experiment by re-testing startle responses of the three DGRP lines used in our study which had the highest startle responses in Mackay et al. (2012), as well as the three lowest lines. This validation was also performed blind to line identity, and full methodological details and results are presented in File S2. We found evidence for interlaboratory variation in male startle response, as expected, but the differences between high and low lines were largely preserved and remained significant (Fig. S1).

### STATISTICAL ANALYSIS

#### Focal male tapping behavior and validation

The initial behavioral dataset described how the incidence of the target behavior—tapping—in focal males depended upon the genotype of their interacting partner. Mixed-model binary logistic regressions were used to assess two questions about whether focal male tapping behavior. The first assessed correspondence between the original and blind validation using the subset of eight lines, and the second examined focal male behavior across all 50 interacting male lines that we screened. In both models, focal male tapping behavior was modeled as a binary response variable, with interacting male line as a random effect and trial temperature as a covariate. In the first, we modeled “block” as a fixed effect because our blocking term only had two factor levels (original vs. validation), which precluded accurate variance estimates. The line × block interaction was included as a random effect, which indicated whether the lines we re-tested responded differently in the initial versus validation experiment. Logit link functions were employed and degrees of freedom were calculated using the Satterthwaite procedure.

To further examine and visualize results from the blind validation, the mean incidence of focal male tapping behavior in the original block was regressed on the mean incidence of focal male tapping behavior in the validation block to assess correspondence between the two. We found a strong positive relationship between the behavior of the eight lines tested in the two blocks (see Results). Given this strong relationship and the results of the above validation analysis, we combined original and validation data for the eight re-analyzed lines. We then examined data from all 50 lines using a second mixed-model binary logistic regression to test whether the DGRP line with which focal males interacted significantly affected their tendency to perform tapping behavior. The latter model included interacting male line as a random effect and temperature as a covariate.

#### Identifying cryptic IGEs

A chief objective of the study was to link any variation in tapping caused by the genotype of interacting partners to identifiable interacting phenotypes. Subsequent analyses therefore tested whether mean phenotype data from the DGRP lines could be used to identify interacting phenotypes causing IGEs on focal male tapping behavior. Line means were used because the phenotype data for chill coma, survival on menadione sodium bisulfite, survival on paraquat, startle response, and starvation resistance were obtained from www.dgrp.gnets.ncsu.edu, whereas the remainder was gathered in this study (orienting, following, tapping, licking, singing, abdomen curling, and general activity). Details of phenotyping methodology for the former are given in Weber et al. (2012) and Mackay et al. (2012). We also tested for an effect of *Wolbachia* infection status of the DGRP lines on the tapping behavior of focal males using a *t*-test to compare the behavior of focal males when paired with infected or noninfected lines. Information on infection status was only available for 43 of the 50 DGRP lines used here. The analysis revealed no difference, so infection status was thereafter disregarded.

Even though focal males were always the same yellow-body strain, we calculated their mean tapping incidence when they were paired with males from each DGRP line. A preliminary analysis used Spearman rank correlation to test whether focal and interacting male tapping behavior were associated, which would suggest a reciprocal IGE. We followed this up with multiple regression using mean focal male tapping behavior as the response variable and mean phenotype values for the DGRP lines as predictors. Data were standardized prior to analysis. Standardizing limits the value of the interaction coefficient ψ from −1 to 1, allowing comparisons of values across studies or taxa (Bleakley and Brodie 2009). Residuals from the regression were normally distributed (Anderson-Darling; *A*^2^ = 0.490, *P* = 0.210), so no further transformations were applied.

Phenotype data were unavailable for some of the 50 lines we used. This resulted in a regression model containing 41 cases with 12 predictors, which caused concern about over-parameterization. We therefore repeated the analysis using only predictor phenotypes where *P* < 0.50. We then repeated the analysis using only predictor phenotypes that were quantified in the present study, enabling the full cohort of 50 DGRP lines to be included. Neither of these procedures changed the results qualitatively, so we only report results from the first analysis. The above statistical analyses were performed using SAS version 9.2 and Minitab version 12.21.

### GENOME-WIDE ASSOCIATION STUDY

A genome-wide association (GWA) study was performed to generate preliminary information about genes or regions underlying IGEs on tapping behavior. The procedure provides a first step for exploring the evolutionary genetics of the IGE; in this case, it bypassed phenotypic information and tested whether any single-nucleotide polymorphisms (SNPs) identified in the DGRP lines was associated with behavioral variation in their interacting partners. We used an online GWA calculator that was custom-built to handle data from the DGRP lines (dgrp.gnets.ncsu.edu).

Briefly, the GWA calculator makes use of nearly 2.5 million SNPs called in the DGRP lines; fewer SNPs are available when less than the full complement of lines are used, as was the case in the present study. In the DGRP lines, major and minor alleles were called for each SNP using criteria described by Mackay et al. (2012) and Harbison et al. (2013): minor alleles had to have been present in at least four lines, and SNPs were only used if coverage was between 2 × and 30 ×. Analysis of variance was performed on each SNP. The line mean phenotype was the response, and each model included a fixed effect of the SNP in question, plus an error term. The marker effect was calculated by dividing the difference in trait values between major and minor alleles by two (Falconer and Mackay 1996, Jordan et al., 2012; Weber et al., 2012).

Potential associated SNPs were highlighted if their *P*-value was 10^−5^ or lower, following published procedures using the DGRP (Jordan et al., 2012; Weber et al., 2012). SNP associations were visualized in a Manhattan plot by implementing the ggplot2 (Wickham 2009) plotting system in R version 2.15.2 (R Development Core Team 2012).

## Results

### FOCAL MALE TAPPING BEHAVIOR AND VALIDATION

**Figure 2:**
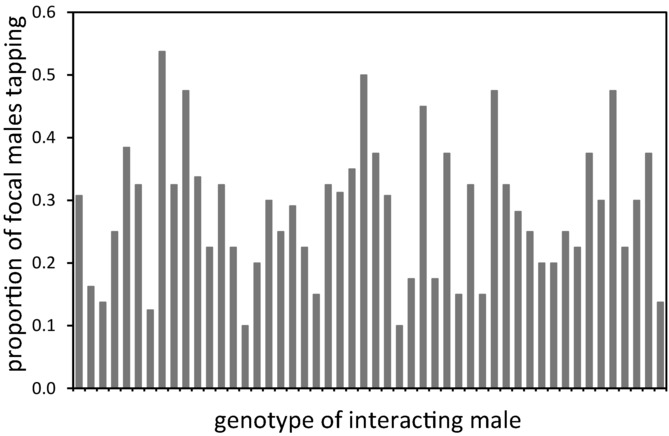
Genotypic variation in focal male tapping behavior. The proportion of focal males exhibiting tapping behavior is shown for each of the 50 inbred lines that were used as interacting partners (*x*-axis). Order of genotypes on the *x*-axis is arbitrary. Interacting male genotype had a significant impact on focal male tapping behavior (see Results for details).

Globally, yellow-body focal males exhibited tapping behavior in 28.16% of all trials, but the incidence of tapping behavior varied considerably depending on the DGRP line with which they were paired (mixed-model binary logistic regression: *n* = 2315, *Z* = 3.21, *P* < 0.001; Fig.2). Depending on the genotype of the interacting male, focal males exhibited tapping in as few as 10% to over 50% of trials (Fig.2). Tapping behavior in focal males did not depend on *Wolbachia* infection status of the DGRP strain with which they interacted (*t*-test: *n* = 43, *t* = 0.57, *P* = 0.57).

**Figure 3:**
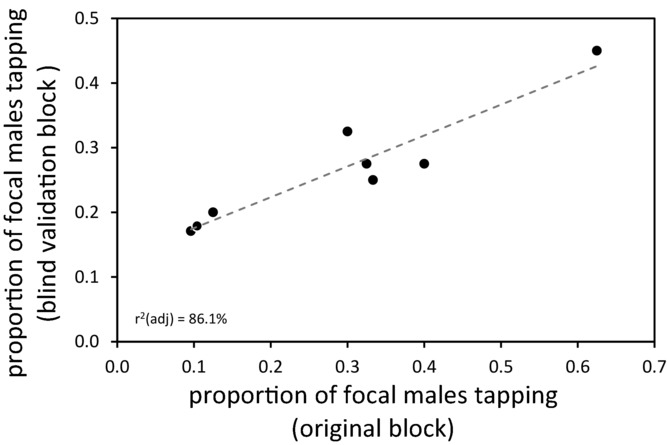
Comparison of blind validation of eight lines to original data from the same lines. Tapping propensity measured in focal flies when the experimenter was blind to the identity of interacting males was associated with tapping propensity measured in focal flies in the original block. The tendency for model males to change their tapping behavior according to the genotype of their partner was thus strongly repeatable. See Results for statistical details, and Table S1 for information about the eight lines used in the blind validation. The best-fit linear regression line is indicated by the gray dashed line. The two points closest to the origin have been jittered as they are exactly overlapping.

Tapping behavior of interacting males was repeatable in a blind validation using a subset of eight of the original DGRP lines. When original data were analyzed with validation data collected under identical conditions but naïve to the identity of interacting male lines, the dependence of focal male behavior on interacting male line was borderline significant (mixed-model binary logistic regression: *n* = 639, *Z* = 1.56, *P* = 0.059), and a nonsignificant interaction between line and block indicated that the tapping IGE was consistent across the two blocks (mixed-model binary logistic regression: *n* = 639, *Z* = 0.10, *P* = 0.458). A linear regression demonstrated the strong positive relationship between the two blocks of data; the effect of interacting genotype on focal male tapping behavior was highly repeatable (linear regression: *r*^2^ (adj.) = 86.1%, *P* = 0.001; Fig.3).

### IDENTIFYING CRYPTIC IGES AND ESTIMATING ψ

**Figure 4:**
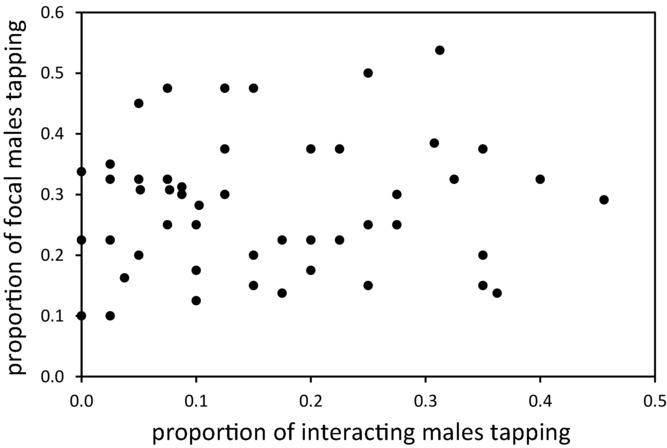
Lack of relationship between focal and interacting male tapping behavior. The incidence of tapping behavior in interacting males did not predict how likely focal males were to exhibit the same behavior so there is no evidence for a reciprocal IGE.

The first analysis to assess whether male tapping behavior has the potential to be a reciprocal interacting phenotype failed support such a scenario (Fig.4). Despite tapping behavior in focal males being highly dependent on the genotype of their interacting partners, there was no relationship between focal and interacting male tapping incidence (Spearman rank correlation: *n* = 50, *r* = 0.048, *P* = 0.741).

**Figure 5:**
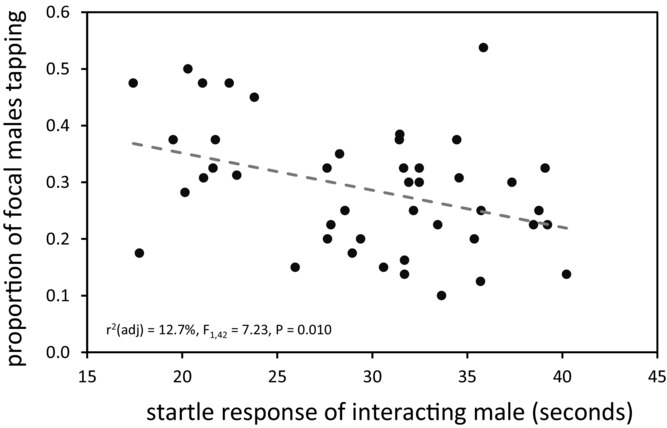
Relationship between startle response of DGRP line males and focal male tapping behavior. This graph shows the only interacting phenotype that predicted focal male tapping behavior with any robustness (see also Table1). Details of how startle response was quantified are given in Weber et al. (2012) and Mackay et al. (2012). Interacting male lines varied in their startle responses, and focal male tapping behavior changed predictably: focal males showed decreased tapping when paired with lines in which flies take longer to settle after being disturbed. The best-fit linear regression line is shown by the gray dashed line.

Multiple regression identified a significant association between startle response of interacting male lines and the incidence of tapping behavior in focal males (Table1). The longer the refractory period of flies that had been startled, the less focal males tapped them (Fig.5). This negative relationship remained significant in a univariate analysis. The mean male startle response of interacting lines explained 12.7% of the variance in tapping behavior in the focal line (linear regression: *n* = 44, *r*^2^ (adj.) = 12.7%, *P* = 0.010). None of the other phenotypes tested showed a relationship with focal male tapping behavior (Table1).

**Table 1:** Multiple regression^2^ examining whether mean phenotypes of interacting male lines predict focal male tapping behavior, and associated estimates of ψ ± standard deviation

Interacting male phenotype	ψ	Standard deviation	*T*	*P*
Chill coma^1^	−0.002	0.172	−0.01	0.990
Survival on MSB^1^	−0.122	0.166	−0.74	0.468
Survival on paraquat^1^	0.237	0.173	1.37	0.182
Startle response^1^	−**0.486**	**0.160**	−**3.03**	**0.005**
Starvation resistance^1^	−0.081	0.163	−0.50	0.624
Orienting	1.299	0.979	1.33	0.195
Following	−0.068	0.474	−0.14	0.886
Tapping	−0.696	0.982	−0.71	0.485
Licking	0.419	0.467	0.90	0.378
Singing	−0.330	0.537	−0.61	0.545
Mounting	−0.352	0.263	−1.34	0.191
General activity	−0.329	0.670	−0.49	0.627

1 Phenotype information consists of male line means from www.dgrp.gnets.ncsu.edu. Details of phenotyping methodology are given in Weber et al. (2012) and Mackay et al. (2012).

The one significant predictor is indicated in bold, and it remained significant after model simplification and individual testing (see Results for details).

2 Full regression model: *r*^2^(adj.) = 25.4%, *F*12,40 = 2.14, *P* = 0.048.

Estimates of ψ ranged from −0.696 to 1.299 (Table1). The single unexpected value where 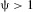 is likely due to the large standard deviation around that particular partial regression coefficient, which did not approach significance in the model (Table1). The estimate of ψ for the only significant interacting male phenotype—startle response—was moderately strong but negative (ψ = −0.486 ± 0.160 SD).

### GWA STUDY

**Figure 6:**
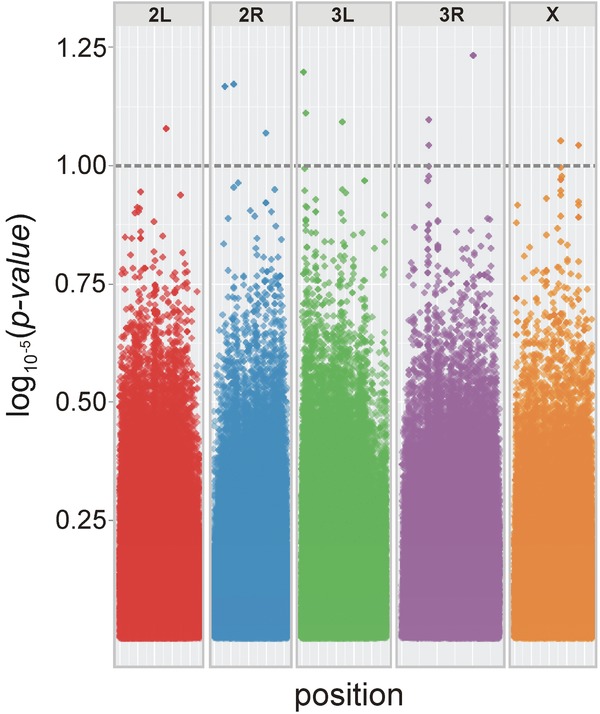
Genome-wide survey for SNPs implicated in IGEs for focal male tapping behavior. Points above the dashed line represent SNPs with a significance level of *P* < 1 × 10^−5^. Some datapoints above the threshold represent more than one SNP position that are located in close proximity; there were 13 significant SNPs in total.

The GWA identified 13 SNPs matching our significance criterion (Fig.6). These were located on all chromosomes, and included SNPs in intronic regions near eight annotated genes. Mean coverage ranged from 6 × to 25 ×, and effect sizes ranged from −0.114 to 0.076. The most significant SNP was located in a 3′ untranscribed region of the protein-coding gene *slowpoke* (*slo*). Other SNPs were located in introns of, or sequences near, the transcription factor *Myocyte enhancer factor 2* (*Mef2*), and the protein-coding genes *Proteasome α6 subunit (Pros α6*), *happyhour* (*hppy*), *Br140*, *Neuropilin and tolloid-like* (*Neto*), *Histidyl-tRNA synthetase* (*Aats-his*), and *Cadherin 87A* (*Cad87A*).

## Discussion

Understanding the evolution of social traits requires detailed information about factors that influence their expression, both genetic and environmental. With respect to the latter, empiricists have long labored over issues such as the roles of learning and imprinting on the ontogeny of social behavior; demographic effects such as density, operational sex ratio, and life history; and the influences of relatedness and population structure (e.g., Alonzo and Sheldon 2010; Hauber and Zuk 2010; Wenseleers et al., 2010). What has become increasingly apparent over the last several decades, however, is that environmental factors influencing the expression of social traits are often found in unexpected places. IGEs transmitted via the social environment, for example, or environmental modifications that organisms themselves create which impact other individuals, can contribute nonintuitively to both the expression and evolution of social traits (Wolf et al., 1998; Odling-Smee et al., 2003; Pelletier et al., 2009; Queller 2011). That the evolutionary implications of such indirect effects have now been recognized, modeled, and documented is encouraging, but the results presented here highlight a persistent difficulty for empiricists: identifying the actual traits involved.

In our *D. melanogaster* study, we found that the tapping behavior of focal males was dependent upon the genotype of their interacting partners. The consistency of the tapping IGE was somewhat surprising given the well-known difficulties of quantifying sensitive behavioral phenotypes, but the repeatability of the IGE in a blind validation confirmed that the effect was not transient or due to sampling error. Despite the apparently strong IGE, the traits of the interacting partners to which focal males responded were cryptic. In other words, we could not initially identify in Figure1A. This underscores a problem. We would predict from a diverse array of theoretical findings that IGEs on tapping behavior have a considerable impact on how tapping behavior evolves (Bailey 2012), but its evolutionary response depends on the types of traits contributing IGEs to it. For instance, if the cryptic IGE was caused by traits in interacting males that are integral to agonistic encounters, such as aggressiveness, it could have important links to the evolution of social dominance (Sartori and Mantovani 2013). If it was caused by traits that contribute to a sexual signal, such as cuticular hydrocarbon components, intersexual selection might proceed at a different rate or produce different equilibrium trait values (Miller and Moore 2007; Bailey and Moore 2012; Rebar and Rodríguez 2013). Similarly, if the cryptic IGE was underpinned by variation in a trait involved in sexual conflict, tapping behavior could theoretically experience diversifying selection (Moore and Pizzari 2005). It was therefore of prime interest to work out the phenotypic underpinnings of this IGE.

We employed two approaches. One was a phenotype-based regression that capitalized on all of the available phenotype information that we had quantified in the 50 DGRP lines we used, plus additional data that had been published previously by other groups. It was developed from trait-based techniques for measuring IGEs (Moore et al., 1997; Bleakley and Brodie 2009; McGlothlin and Brodie 2009). At the end of our analysis, the only DGRP phenotype that bore any relation to the tapping behavior of focal males was male startle response, which had a significant interaction coefficient of ψ = −0.486. The strength and direction of this IGE was intuitive. Focal males were less likely to tap interacting males that took longer to alight after having been disturbed, which stands to reason as there would be fewer opportunities to approach a moving male and make physical contact with him. It is also possible that an interacting male's startle response affected the rate of focal male tapping when they were in contact, although this seems less likely given that our measure of tapping was based on the incidence, not the intensity, of the behavior. An additional experiment provided evidence that DGRP line-specific variation in male tapping behavior is broadly repeatable across laboratory environments (File S2), adding a measure of confidence to conclusions based on DGRP data collected by other groups.

Our analysis underscores a logistical constraint in any quest to identify a cryptic IGE, which is that it is not feasible to regress a focal phenotype on an infinite number of interacting partner phenotypes to identify promising candidates. Nevertheless, long-term, large-scale empirical studies are being performed in a number of laboratory model organisms and field systems which enables researchers to capitalize on increasingly rich, multidimensional repositories of phenotypic data. In addition to the DGRP (Mackay et al., 2012), examples include *Drosophila pseudoobscura* sexual selection lines (Hunt et al., 2012); long-term field studies of Soay sheep (*Ovis aries*: Clutton-Brock and Pemberton 2004), song sparrows (*Melospiza melodia*: Reid 2012), collared flycatchers (*Ficedula albicollis*: Björklund et al. 2013), and field crickets (*Gryllus campestris*: Rodríguez-Muñoz et al. 2010); plus experimental evolution studies using microbes such as *Escherichia coli* (Lenski and Travisano 1994), yeast (*Saccharomyces cerevisiae*: Samani and Bell 2010), and viruses (bacteriophage Φ2: Leggett et al., 2013). It may therefore be feasible in more systems than are currently appreciated for researchers to utilize such data resources to identify IGEs contributing to social evolution. We anticipate that such an approach would reveal surprising evolutionary links between seemingly disparate phenotypes, in the same way that our analysis above suggests an intuitive yet unforeseen relationship between tapping behavior and startle response in *D. melanogaster*.

The second approach we took circumvented the phenotype Z' to directly assess genomic regions underlying the cryptic tapping IGE. As with our phenotypic analysis, the GWA is necessarily a first step in identifying promising candidate genomic locations or genes, and follow-up work is required to validate and assess any candidate genes with hints of indirect effects. There is some controversy regarding the use and interpretation of GWA approaches (Marjoram et al., 2014), particularly with regard to establishing threshold levels of significance for associated SNPs. However, our aim is to establish a framework that can be used to probe the identity of strong but cryptic IGEs, by suggesting promising phenotypic and genetic candidates, rather than establishing definitive proof at this stage.

Using the SNP dataset available for the DGRP lines, we were able to identify approximately a dozen protein-coding genes that might affect the expression of tapping behavior in focal males. Variation at these sites may potentially play a causal role in the IGE for tapping behavior (represented by in Fig.1A and C). It is of note that the most significantly associated gene was *slowpoke* (*slo*). Some of the behavioral phenotypes associated with *slo* mutants include decreased flight ability and a “sticky-feet” phenotype in which affected flies appear unable to move from a stationary position, as if their feet were adhered to the substrate (Atkinson et al., 2000; Brenner et al., 2000). The “sticky-feet” effect associated with *slo* is consistent with a link to the startle response of interacting males, and it suggests a promising candidate locus underlying the tapping IGE.

Identifying genetic variants associated with complex behavioral phenotypes is a topical challenge in evolutionary, behavioral, and medical genetics, and there is debate regarding the merit of attempting to resolve the genetic architecture of polygenic traits into effects at individual loci using genomic association studies (Travisano and Shaw 2013). We suggest, therefore, that results such as ours provide a starting point for identifying not only genes that may play pivotal roles in causing IGEs, but also a foundation for characterizing functional and epistatic networks underlying those IGEs, as is becoming increasingly common in this and other study systems (Stern and Orgogozo 2009; Swarup et al., 2013). The SNPs we identified in our GWAS by no means reveal the definitive gene(s) underlying the tapping IGE in *D. melanogaster*. However, they provide tantalizing clues linking genes, interacting phenotypes, and the focal tapping behavior, and they warrant future investigation.

Studying IGEs as multivariate traits provides an alternative, or at least complementary, approach for identifying and characterizing those that are cryptic. The quantitative genetics framework that was developed to model and predict effects of IGEs in the 1990s was readily extended to multivariate trait evolution (McGlothlin et al., 2010). The effect of IGEs on multivariate trait evolution is captured by slightly more complex matrices of interaction coefficients describing the pattern of IGEs on all traits under consideration: 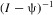 (Moore et al., 1997) and feedback effects generated by reciprocal IGEs: 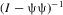 (McGlothlin et al., 2010). It is possible to test and estimate IGEs for multiple traits at once, as has been done to investigate IGEs on male cuticular hydrocarbon components in *D. serrata* (Petfield et al., 2005). Using a multivariate approach also affords the opportunity to characterize multicomponent interacting phenotypes, for example, by describing interacting phenotypes as principal components in multivariate trait space, which potentially avoids some of the pitfalls associated with trying to pinpoint univariate traits.

Ultimately, the challenge of identifying cryptic IGEs stands regardless of whether a univariate or multivariate approach is used. There is a fine distinction to be made between studies that focus on the potential for IGEs to affect the expression of traits, for example, assessing the likelihood of reciprocal IGEs in intrasexual aggression, and studies that are concerned with how a particular social trait evolves, for example, whether IGEs of any description affect sexual selection via female choice. Cryptic IGEs are more likely to hinder progress on the latter. However, we suggest the number of systems that can use large-scale, top-down approaches to identify such IGEs may not be so few. In addition, future development of genomic resources may make it easier to work from the opposite direction: by identifying genomic regions that are directly implicated in IGEs, the putative function and roles of those genes might provide clues as to the interacting phenotypes involved, ultimately allowing researchers to causally link genes with the evolution of interacting phenotypes.

## DATA ARCHIVING

Behavioral data collected for this study (orienting, following, tapping, licking, singing, abdomen curling, general activity, and startle response) have been archived on Dryad (doi:10.5061/dryad.d4s1k). Line means for chill coma, survival on menadione sodium bisulfite, survival on paraquat, startle response, and starvation resistance were obtained from www.dgrp.gnets.ncsu.edu as described in Mackay et al. (2012) and Weber et al. (2012). Genomic data (reads and SNPs) are publicly archived at http://dgrp.gnets.ncsu.edu/.

## Supporting Information

Disclaimer: Supplementary materials have been peer-reviewed but not copyedited.

Table S1 DGRP lines used in the current study and the incidence of tapping behaviour performed in each line, plus the incidence of focal male tapping behavior when paired with each line.

Figure S1 Male startle response in six re-tested RAL lines, comparing original published phenotype data (Mackay et al. 2012) with data collected in the present study.
