# Supplementary material for: DETECTING CRYPTIC INDIRECT GENETIC EFFECTS
Source: Evolution. 2014 May 5;68(7):1871–82. doi: 10.1111/evo.12401 (PMC4257566; doi:10.1111/evo.12401)
Supplement: Table S1 — DGRP lines used in the current study and the incidence of tapping behaviour performed in each line, plus the incidence of focal male tapping behavior when paired with each line. [file evo0068-1871-SD1.docx]

**Supplementary Table S1. DGRP lines used in the current study and the incidence of tapping behaviour performed in each line, plus the incidence of focal male tapping behaviour when paired with each line.**

| **DGRP line** | **Tapping incidence in DGRP line** | **Tapping incidence in focal line** |
| --- | --- | --- |
| 21 | 0.0513 | 0.3077 |
| 38* | 0.0375 | 0.1625 |
| 75* | 0.3625 | 0.1375 |
| 85 | 0.1000 | 0.2500 |
| 88 | 0.3077 | 0.3846 |
| 93 | 0.3250 | 0.3250 |
| 136 | 0.1000 | 0.1250 |
| 149* | 0.3125 | 0.5375 |
| 158 | 0.0500 | 0.3250 |
| 208 | 0.1500 | 0.4750 |
| 223* | 0.0000 | 0.3375 |
| 228 | 0.2000 | 0.2250 |
| 239 | 0.0250 | 0.3250 |
| 301 | 0.2250 | 0.2250 |
| 303 | 0.0250 | 0.1000 |
| 315 | 0.3500 | 0.2000 |
| 321* | 0.0875 | 0.3000 |
| 324 | 0.2750 | 0.2500 |
| 336* | 0.4557 | 0.2911 |
| 358 | 0.0250 | 0.2250 |
| 360 | 0.2500 | 0.1500 |
| 365 | 0.4000 | 0.3250 |
| 373* | 0.0875 | 0.3125 |
| 375 | 0.0250 | 0.3500 |
| 379 | 0.2500 | 0.5000 |
| 380 | 0.3500 | 0.3750 |
| 392 | 0.0769 | 0.3077 |
| 398 | 0.0000 | 0.1000 |
| 405 | 0.2000 | 0.1750 |
| 406 | 0.0500 | 0.4500 |
| 426 | 0.1000 | 0.1750 |
| 443 | 0.1250 | 0.3750 |
| 491 | 0.3500 | 0.1500 |
| 502 | 0.0750 | 0.3250 |
| 530 | 0.1500 | 0.1500 |
| 535 | 0.1250 | 0.4750 |
| 584 | 0.0750 | 0.3250 |
| 639 | 0.1026 | 0.2821 |
| 738 | 0.2500 | 0.2500 |
| 757 | 0.1500 | 0.2000 |
| 783 | 0.0500 | 0.2000 |
| 801 | 0.0750 | 0.2500 |
| 802 | 0.0000 | 0.2250 |
| 805 | 0.2250 | 0.3750 |
| 808 | 0.2750 | 0.3000 |
| 812 | 0.0750 | 0.4750 |
| 850 | 0.1750 | 0.2250 |
| 884 | 0.1250 | 0.3000 |
| 890 | 0.2000 | 0.3750 |
| 897* | 0.1750 | 0.1375 |

***Lines that were used in the blind validation assay. For these eight lines, the incidence of tapping behaviour reported in the table represents the combined incidence across both the original and validation assays (see Results).**
