# Supplementary material for: DETECTING CRYPTIC INDIRECT GENETIC EFFECTS
Source: Evolution. 2014 May 5;68(7):1871–82. doi: 10.1111/evo.12401 (PMC4257566; doi:10.1111/evo.12401)
Supplement: Figure S1 — Male startle response in six re-tested RAL lines, comparing original published phenotype data (Mackay et al. 2012) with data collected in the present study. [file evo0068-1871-SD2.docx]

**Supplementary Data: Startle Response Validation**

**
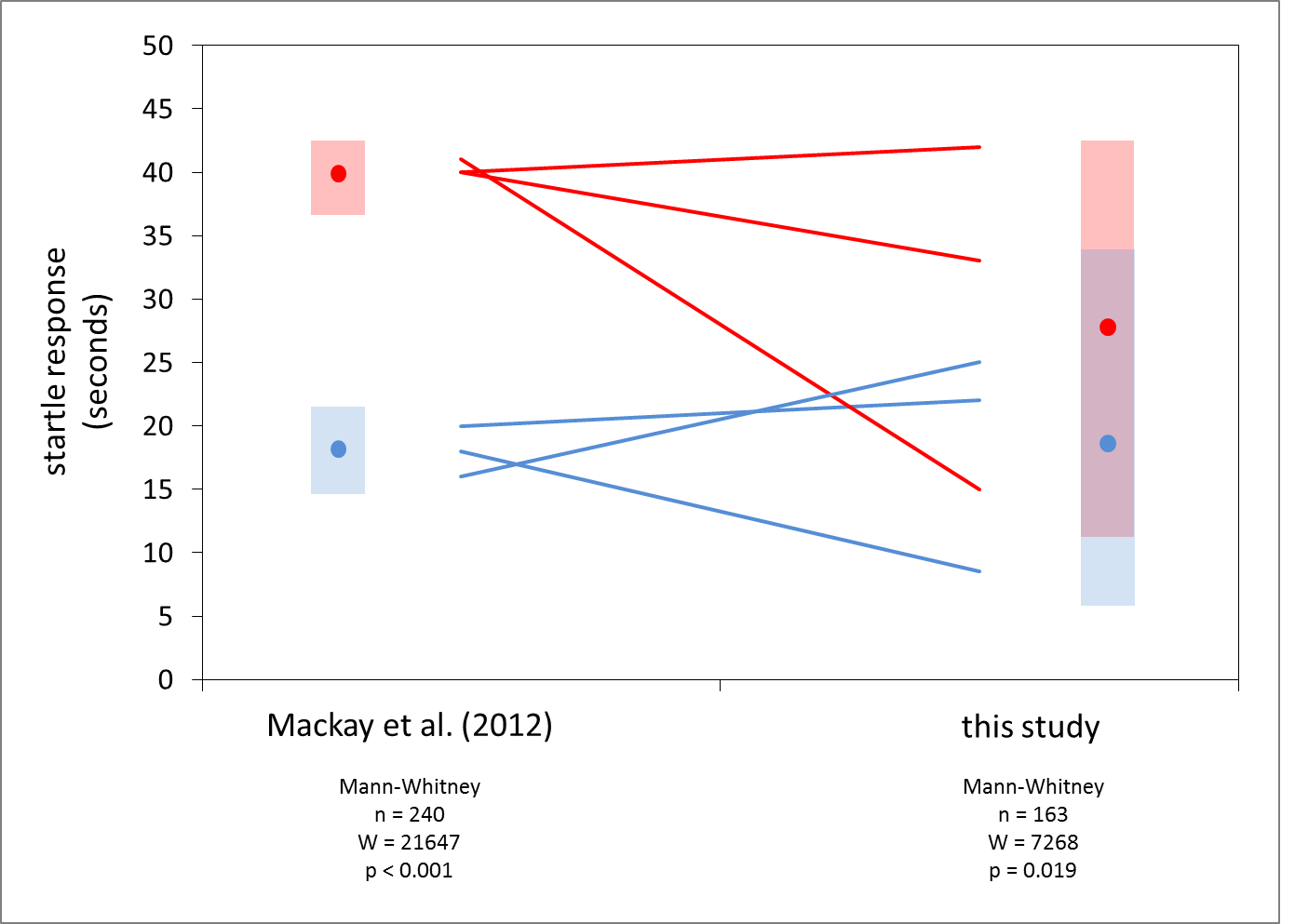
**

**Figure S1. Male startle response in six re-tested RAL lines, comparing original published phenotype data (Mackay et al. 2012) with data collected in the present study.** Values for the overall comparison between “high” and “low” startle response lines are indicated by solid dots (medians) and shaded boxes (interquartile ranges) to either side of the graph. Results from separate Mann-Whitney tests comparing “high” and “low” lines are indicated, and median startle responses for each of the six-retested lines are calculated from the publicly available data described in Mackay et al. (2012) and the present study. Interquartile ranges for the lines overlapped considerably and are therefore not shown.

**Methods and Results Summary**
Our experimental protocol replicated Jordan et al. (2007) and Mackay et al. (2012) as faithfully as possible. We selected the three RAL lines used in the present study that showed the highest startle response in Mackay et al. (2012) (RAL_897, RAL_358, RAL_93), as well as the three lowest (RAL_353, RAL_405, RAL_805). The experiment was performed blind to fly line identity until after all data were collected.

Stock flies were reared in large vials (29mm x 95mm) on standard cornmeal agar medium seeded with yeast. All flies experienced a 12h:12h light:dark cycle at 25 °C. We tipped adults between 0 and 4 days old into new vials to ensure the proper age range of test subjects, and then two days later isolated males under light CO_2_ into individual small vials (16mm x 95mm) with approximately 5 mL of cornmeal agar medium. They were allowed to rest for 24 hours before testing. All flies were thus between 3 and 7 days old at the time of testing.

On the day of testing, we assessed startle response: two hours after the onset of the flies’ light cycle (08:00), each individual’s vial was gently tapped on the surface of the laboratory bench and the total amount of time that the fly was mobile (flying or walking) in the following 45 seconds was recorded as the startle response. Tests were performed at 25 °C under artificial light, and the order in which the different blind-coded lines were tested was randomised. All flies were tested on the same day.

Visual inspection of the data confirmed the expected inter-lab variation, in particular, startle response data from our study had notably greater variance. This may be partly due to the smaller number of flies we tested, but unaccounted methodological differences or other environmental factors are probable as well. Nevertheless, our data broadly support the repeatability of startle response variation found by Mackay et al. (2012), recovering a significant difference in the expected direction between “high” and “low” lines (Figure S1), with only one line out of the 6 switching from “high” to “low”.

Because our data were distributed non-normally and could not be transformed to approach normality, plus the fact that variance was substantially higher in our dataset, we performed separate Mann-Whitney U tests comparing “high” and “low” lines in both the publicly-available phenotype data from Mackay et al. (2012) and our own data. Analyses were performed in Minitab v.12.21 and are presented above (Figure S1). With the exception of one “high” line, those that were found to have high startle responses in Mackay et al. (2012) remained high in our study, and vice versa.

**Literature Cited**

Jordan, K. W., K. L. Craver, M. M. Magwire, C. E. Cubilla, T. F. C. Mackay, and R. R. H. Anholt. 2012. Genome-wide association for sensitivity to chronic oxidative stress in *Drosophila melanogaster*. PLoS ONE 6:e38722.

Mackay, T. F. C., S. Richards, E. A. Stone, A. Barbadilla, J. F. Ayroles, D. Zhu, S. Casillas, Y. Han, M. M. Magwire, J. M. Cridland, M. F. Richardson, R. R. H. Anholt, M. Barrón, C. Bess, K. P. Blankenburg, M. A. Carbone, D. Castellano, L. Chaboub, L. Duncan, Z. Harris, M. Javaid, J. C. Jayaseelan, S. N. Jhangiani, K. W. Jordan, F. Lara, F. Lawrence, S. L. Lee, P. Librado, R. S. Linheiro, R. F. Lyman, A. J. Mackey, M. Munidasa, D. M. Muzny, L. Nazareth, I. Newsham, L. Perales, L. –L. Pu, C. Qu, M. Rámia, J. G. Reid, S. M. Rollmann, J. Rozas, N. Saada, L. Turlapati, K. C. Worley, Y. –Q. Wu, A. Yamamoto, Y. Zhu, C. M. Bergman, K. R. Thronton, D. Mittelman, and R. A. Gibbs. 2012. The *Drosophila melanogaster* genetic reference panel. Nature 482:173-178.
